# Soluble HLA-G expression levels and HLA-G/irinotecan association in metastatic colorectal cancer treated with irinotecan-based strategy

**DOI:** 10.1038/s41598-020-65424-z

**Published:** 2020-05-29

**Authors:** Lucia Scarabel, Marica Garziera, Sara Fortuna, Fioretta Asaro, Giuseppe Toffoli, Silvano Geremia

**Affiliations:** 1 Experimental and Clinical Pharmacology Unit, Centro di Riferimento Oncologico (CRO), IRCCS, 33081 Aviano, Italy; 2 Department of Chemical and Pharmaceutical Sciences, University of Trieste, Via L. Giorgieri 1, 34127 Trieste, Italy

**Keywords:** Colorectal cancer, Computational chemistry, Receptor pharmacology, Pharmacokinetics, Chemotherapy

## Abstract

We here explore the soluble Human Leukocyte Antigen-G (sHLA-G) expression level as clinical biomarker in metastatic colorectal cancer (mCRC). To this aim the sHLA-G protein was measured in plasma samples of 40 patients with mCRC treated with the FOLFIRI (irinotecan (CPT-11) plus 5-fluorouracil (5-FU) and leucovorin (LV)) regimen. The results suggest a link between HLA-G levels and irinotecan (CPT-11) pharmacokinetic, leading to hypothesize a molecular interaction between sHLA-G and CPT-11. This interaction was confirmed experimentally by fluorescence spectroscopy. HLA-G is known to exist in a number of polymorphs that affect both the protein expression levels and its peptide-binding cleft. The interaction between HLA-G polymorphs and CPT-11 was explored by means of computational modelling, confirming the hypothesis that CPT-11 could actually target the peptide binding cleft of the most common HLA-G polymorphs.

## Introduction

The possibility to measure and monitor the level of soluble Human Leukocyte Antigen-G (sHLA-G) in body fluids such as plasma, serum, ascites, cerebrospinal fluids exudates, and the identification of early variations in HLA-G levels is emerging as a precious tool for the early diagnosis of cancer diseases and to monitor the appearance of recurrences^1^. Although tissue and/or soluble HLA-G were already observed upregulated in patients with several cancers compared to healthy donors^2–6^, further investigation about its therapeutic role is still needed.

HLA-G exerts immunoinhibitory activities mainly through a direct interaction with ILT receptors on effector cells and an indirect mechanism through regulatory cells^7^. A specific inhibitory interaction of HLA-G expressed by target cells against TILs, in particular CD8+ ILT2+ T cell cytotoxicity, was reported as a mechanism of tumor escape from immunosurveillance^8^. Recently, Pagès and colleagues showed the relevance of a new method called Immunoscore to stratify patients evaluating the presence of CD3+ and CD8+ T-cell effectors (TILs) and ensuring a more precise treatment with a better prognostic value in colon cancer^9^. However, not all the independent evaluators are concordant for the TIL evaluation on hematoxylin and eosin stained and the concordant analysis was performed only on 10% of whole cohort (n = 268 cases)^9^.

The clinical relevance of sHLA-G expression has recently suggested that a stratification based on sHLA-G levels could also be an independent predictive and prognostic factor for patients with colorectal cancer (CRC) because its presence could alter the TILs interaction and then the immune system response^10^. Moreover, these patients are often administered with irinotecan (CPT-11) as part of their therapy. CPT-11 is an antineoplastic drug present in several regimens used in combination with other chemotherapeutics to treat metastatic CRC (mCRC). It is also investigated for clinical applications in gastric, lung, pancreas, cervix, and ovarian cancers^11–16^. CPT-11 acts as antineoplastic enzyme inhibitor of the DNA topoisomerase I, interfering with the replication of the DNA and inducing double-breaks in the strand, causing cell death^17,18^. Its therapeutic efficacy is partially reduced by the occurrence of severe toxicities, such as diarrhea and neutropenia, that limits the irinotecan clinical use^19^. Research of potential genetic biomarkers to reduce or control irinotecan toxicity profile, was mainly focused on metabolic enzymes: in fact, to date, UGT1A1*28 polymorphism is the only validated pharmacogenetic biomarker recommended by FDA guidelines for a reduction of the starting dose of irinotecan^20^. Irinotecan has a highly complex metabolism^21^. UGT1A1*28 polymorphism causes an impaired detoxification of the 7-ethyl-10-hydroxycamptothecin (SN38), the active metabolite of CPT-11, to the inactive SN38 glucuronide (SN38G) leading to increase toxicities and possible alteration in the efficacy of treatment. Moreover, an alternative pathway of CPT-11 detoxification is due to cytochrome-mediated oxidation in inactive metabolite (NPC and APC) that could be converted into SN38 by carboxylesterase as well. Several techniques has been developed for the quantification of the irinotecan and its metabolites and also for the simultaneous and rapid quantification of different chemotherapeutic drugs used for the treatment of patients with different kind of cancers^22,23^. Here we suggest that also an immunocheckpoint molecule like the HLA-G could be an interesting candidate biomarker for patients with mCRC treated with irinotecan-containing regimens.

**Figure 1:**
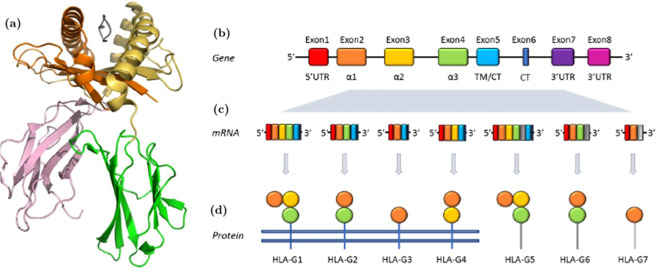
HLA-G and its main isoforms. (**a**) Crystal structure (PDB ID: 1YDP) of the extracellular HLA-G complex α1 (orange), α2 (yellow), and α3 (green colour) globular domains non-covalently associated with β2-microglobulin (pink colour) and antigen peptide (grey). (**b**) Exon 1 codifies for the 1–24 aa peptide signal that is lost in the mature protein (red), exon 2 generates the α1 domain, exon 3 the α2 domain (yellow), exon 4 the α3 domain. Exons 5 (light blue) and 6 for the transmembrane (TM) and cytoplasmic (CT) domains, respectively. (**c**) HLA-G mRNA transcripts and (**d**) resulting proteins.

The *HLA-G* gene encodes the HLA-G protein, a non-classical major histocompatibility complex (MHC) class I molecule (Fig. 1a,b)^24,25^. As the result of alternative splicing on its primary mRNA (Fig. 1c) HLA-G can exist in a number of isoforms^7,26–29^, with the most characterized to date (schematised in Fig. 1d) being four membrane bound (HLA-G1 to -G4) and three soluble (HLA-G5 to -G7) generated by the retention of a stop codon after exon 4, all capable of exerting a negative regulation on immune cells^7^. Further, HLA-G1 isoform may exists as soluble “shed” HLA-G1 (sHLA-G1) through proteolytic cleaving from the cell surface^2^ suggesting that ectopic expression could have a clinical significance, in particular in the progression of different malignancies^1^. All the HLA-G isoforms reported in Fig. 1d contains the α1 domain. However, the HLA-G2/6 and HLA-G3/7, by lacking the α2 domain, are not able to present antigens. This exemplifies one of the peculiarities of the non-classical HLA-G functions.

Anyhow, even when both the α1 and α2 domains are present, the existence of *HLA-G* coding nonsynonymous polymorphisms is still expected to affect both the peptide-binding cleft and the availability of the circulating protein.

In the general population, contrary to classical (class Ia) MHC molecules, the nonclassical HLA-G (class Ib) is characterized by a limited allelic variation, in particular, to date only 69 alleles of HLA-G have been identified (see http://hla.alleles.org/data/hla-g.html, Release 3.38.0, 17 October 2019), which encode 19 transmembrane proteins (HLA-G*01:01 to HLA-G*01:04, HLA-G*01:06 to HLA-G*01:12, HLA-G*01:14 to HLA-G*01:20 and HLA-G*01:22) and 3 truncated/null proteins (HLA-G*01:05 N, HLA-G*01:13 and HLA-G*01:21). The HLA-G*01:01 protein allele is the most prevalent in different European populations then considered as reference allele, followed by the HLA-G*01:04, HLA-G*01:06, HLA-G*01:03 and HLA-G*01:05 N^30^. Further, several studies have reported an association between sHLA-G levels and specific polymorphisms in coding HLA-G alleles and in the non-coding regions. Specifically, regarding polymorphisms affecting the coding regions, the HLA-G*01:04 and the HLA-G*01:05 N have been associated with high and low sHLA-G levels, respectively^2,31,32^. Polymorphisms in the untranslated/non-coding regions have been also reported to influence the amount of secreted sHLA-G^33–39^, the survival as well the risk of develop CRC^40,41^, and the onset of severe toxicity after chemotherapy treatment in patients with CRC^42^.

Here we studied the association between the sHLA-G levels in plasma samples and clinical characteristics of patients with mCRC and irinotecan (CPT-11) pharmacokinetic parameters. The possible molecular interactions between HLA-G and CPT-11 was tested *in vitro* by UV-Vis spectrophotometry and fluorescence spectroscopy, and the interaction between HLA-G polymorphs (or mutants) and CPT-11 was explored *in silico* by means of docking and molecular dynamics simulations.

## Results

### Clinical parameters and soluble HLA-G plasmatic levels

Untreated plasma and blood samples collected from 40 patients with mCRC prior to administration of FOLFIRI (irinotecan (CPT-11) plus 5-fluorouracil (5-FU) and leucovorin (LV)) regimen, were considered for this analysis. In this group (Table 1), with predominance of women (*n* = 25, 62.5%) and of rectum and right colon as primary tumor site (*n* = 18, 45.0% and *n* = 14, 35.0%, respectively), the median age was 57.9 years (range 26.5–75.5) and median follow-up was 15.42 months (range: 1.15–31.48 months). Most of the patients with mCRC were staged III-IV at the time of diagnosis (*n* = 35, 87.5%), received radical surgery (*n* = 26, 65.0%), and 8 out of 40 (20.0%) patients had more than 2 metastatic sites at the time of enrolment in the study.

**Table 1:** Demographic and clinical characteristics of 40 eligible patients with mCRC and association of sHLA-G plasma expression with their clinicopathological parameters.

Characteristic	*n*	(%)	sHLA-G median (range, U/ml)	p*
*mCRC*			116.4 (12.8–1552.7)	
*Age, yrs*				
≤61.6	20		117.9 (43.1–1552.7)	0.8604
>61.6	20		116.4 (12.8–806.0)	
*Gender*				
Woman	25	62.5	121.4 (43.1–1552.7)	0.1464
Man	15	37.5	91.5 (12.8–254.5)	
*Primary tumor site*				
Colon	22	55.0	110.04 (12.8–199.7)	0.5054^a^
Rectum	18	45.0	123.6 (43.1–1552.7)	
Left colon	8	20.0	113.8 (12.8–199.7)	0.8112
Right colon	14	35.0	94.2 (44.4–806.0)	
*Radical surgery*				
Yes	26	65.0	125.8 (48.4–806.0)	0.1605
No	14	35.0	89.3 (12.8–806.0)	
*Stage at diagnosis*				
I-II	5	12.5		
III-IV	35	87.5		
*Adjuvant (radiotherapy or chemotherapy)*				
No	26	65.0	102.0 (12.8–1552.7)	0.6000
Yes	14	35.0	123.6 (61.8–366.5)	
*Number of metastatic sites*				
1	17	45.9	84.1 (43.1–711.4)	**0.0214**
>1	20	54.1	150.1 (12.8–1552.7)	
2	12	32.4		
3	2	5.4		
4	5	13.5		
5	1	2.7		
>2	8	21.6		**0.0486**

*Mann-Whitney-test; ^a^*p *= 0.759 with Kruskal-Wallis test comparing rectum, left colon and right colon.

**Figure 2:**
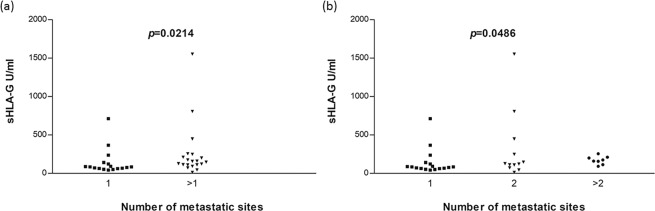
Statistical comparison (**a**) between 1 metastatic site and>1 metastatic site based on Mann-Whitney test, and (**b**) between 1, 2 and >2 metastatic site based on Krustal Wallis test.

To measure the sHLA-G a commercially available ELISA kit was used. The median sHLA-G was 116.4 U/ml (range: 12.8–1552.7). The clinicopathological parameters of the patients with mCRC were stratified according to the sHLA-G plasma levels measured (Table 1). No significant associations were observed except for the number of metastatic sites. Significantly lower sHLA-G levels (*p* = 0.0214, Fig. 2a) were observed in patients with 1 metastatic site (median 84.1 U/ml) compared with patients with more than 1 metastatic site that were associated with a higher sHLA-G concentration (median 150.1 U/ml). This result was also confirmed (*p* = 0.0486) when patients were stratified according to 1 metastatic site, 2 metastatic sites and more than 2 (>2) extra-regional lesions (Fig. 2b).

### Correlation between sHLA-G levels and pharmacokinetic parameters

**Figure 3:**
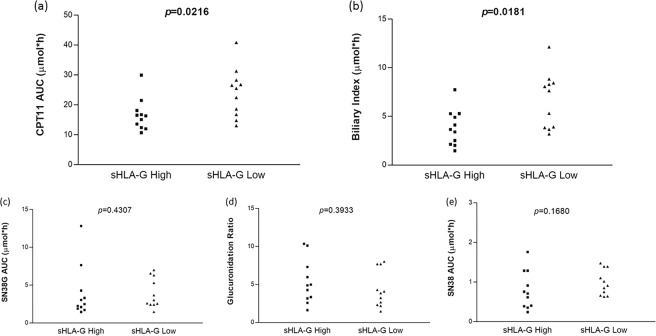
Distribution of pharmacokinetic parameters according to plasma sHLA-G High or Low group: (**a**) CPT-11 AUC, (**b**) biliary index, (**c**) SN38 AUC, (**d**) SN38G AUC, (**e**) glucuronidation ratio.

Based on the possible role of the sHLA-G plasmatic level in patients with mCRC treated with first-line FOLFIRI regimen, we further investigated the possible correlation between sHLA-G levels and pharmacokinetic parameters (PK) previously reported by our group^43^ CPT-11 AUC, SN38 AUC (the CPT-11 active form), SN38G (inactive glucuronidation form of irinotecan), GR (glucuronidation ratio) and BI (biliary index). The BI was defined as the product of the CPT-11 AUC and the ratio of the SN38 AUC over the SN38G AUC: BI = [AUC(CPT11)]*[AUC(SN38)/AUC(SN38G)]. For this analysis, PK values were available for 22 patients that were stratified into two subgroups according to the sHLA-G median value (86.4 U/ml) measured: above (sHLA-G High) and equal or below (sHLA-G Low) the median sHLA-G value. Patients with sHLA-G High were associated with lower levels of plasmatic CPT-11 (median 16.3 µmol*h, range: 10.7–29.9) and patients with sHLA-G Low with higher levels of CPT-11 (median 25.5 µmol*h, range: 13–40.8) (*p* = 0.0216, Fig. 3a). Similarly, a significant inverse correlation was found between sHLA-G and BI (*p* = 0.0181, Fig. 3b): patients with sHLA-G High were associated with lower levels of BI (median 3.6 µmol*h, range: 1.4–7.7) and patients with sHLA-G Low with increased levels of BI (median 7.6 µmol*h, range: 3.2–12.1). No correlation was found with SN38 AUC, SN38G, or GR (Fig. 3c–e).

### HLA-G/irinotecan interaction

The inverse relation observed between sHLA-G and CPT-11 AUC levels suggested an irinotecan-based effect on sHLA-G. We thus explored the possible molecular interactions between HLA-G and irinotecan *in vitro* and *in silico*.

**Figure 4:**
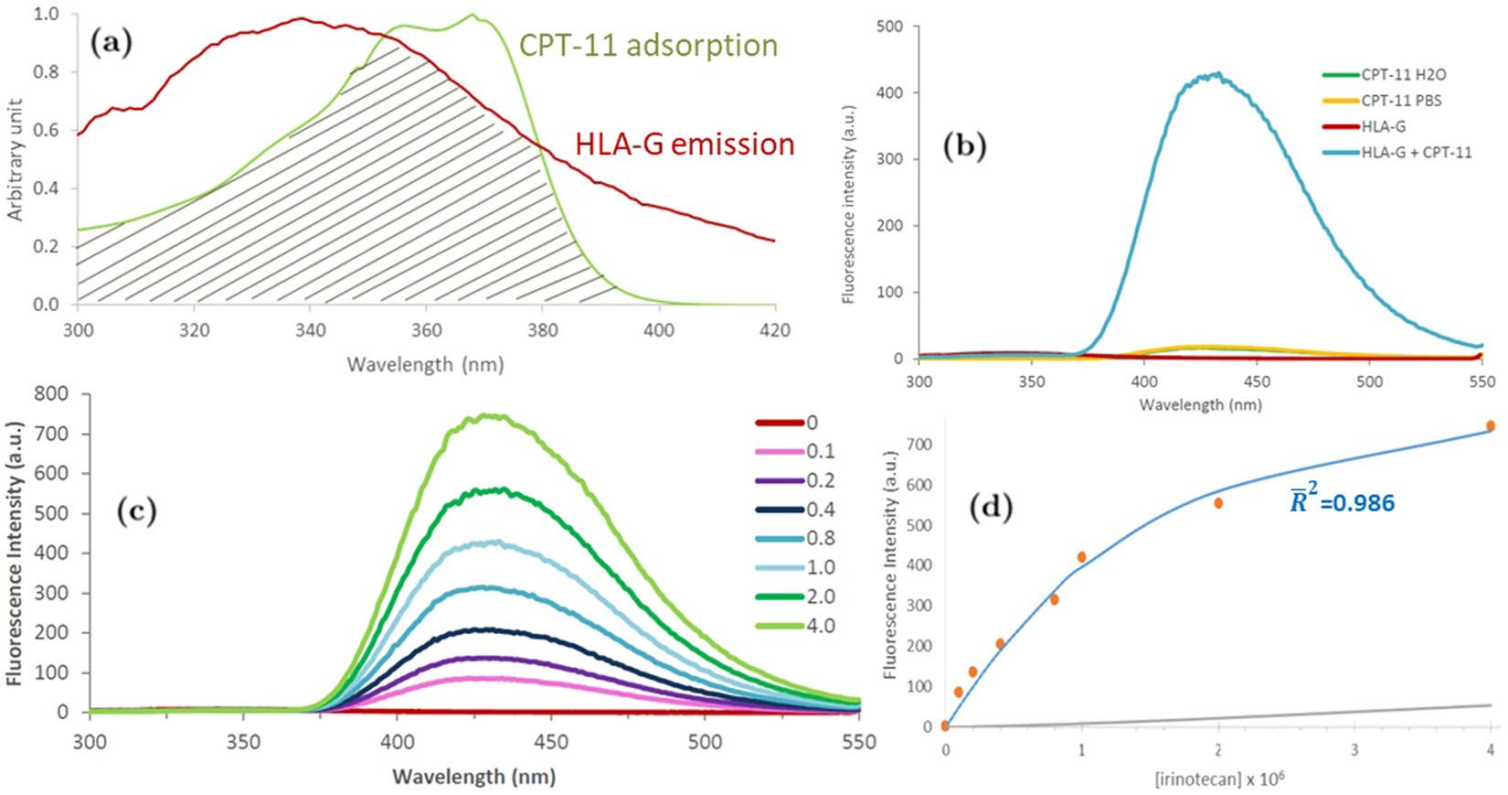
(**a**) Overlap (shaded area) between absorbance spectrum of CPT-11 50 μM (green) and emission spectrum of HLA-G 1 μM (red) suggesting FRET. (**b**) Emission spectra of CPT-11 in water (green), CPT-11 in 1 μM PBS (yellow), 0.1 μM HLA-G (red), and HLA-G 1 μM with CPT-11 1 μM (ratio 1:1) in water (blue). (**c**) Fluorescence emission spectra (mean of 4 scans) of HLA-G 1 μM with the addition of CPT-11 at 0.1, 0.2, 0.4, 0.8, 1, 2, 4, 8 μM. (**d**) Comparison between the free irinotecan (gray line) and the fit (blue curve) to the experimental fluorescence data (red points) at 430 nm of panel c. The adjusted coefficient of determination (${\bar{{\boldsymbol{R}}}}^{2}$) relative to the fitted curve is also indicated. Errorbars are smaller than datapoint sizes. All the emission spectra were recorded with an excitation wavelength of 280 nm.

The interaction between HLA-G*01:01 and CPT-11 was experimentally investigated by spectrophotometric analysis. The emission spectra of the HLA-G overlaps considerably with the absorption spectra of CPT-11 (Fig. 4a) suggesting the possibility to use the fluorescence resonance energy transfer (FRET) between HLA-G and CPT-11. The measurements were performed in water solutions because no appreciable variation in the shape and intensity of CPT-11 emission spectra was observed in PBS (Fig. 4b). A huge variation of the fluorescence intensity, compared to the signals produced by the HLA-G and CPT-11 alone, was observed when the HLA-G and CPT-11 solutions were mixed in a 1:1 ratio (Fig. 4b). The large increase of the fluorescence signal attributable to the FRET confirmed the interaction between the HLA-G and CPT-11. A titration assay further confirmed the dose response signal with a significant increase of the CPT-11 fluorescence (Fig. 4c). The fit of the datapoints collected at 430 nm (Fig. 4d) allowed to estimate the HLA-G/CPT-11 dissociation constant (Kd = 0.54 ± 0.17 µM). The result, calculated as detailed in Supporting Information, was validated with adjusted coefficient of determination ${\bar{R}}^{2}$ = 0.986.

**Figure 5:**
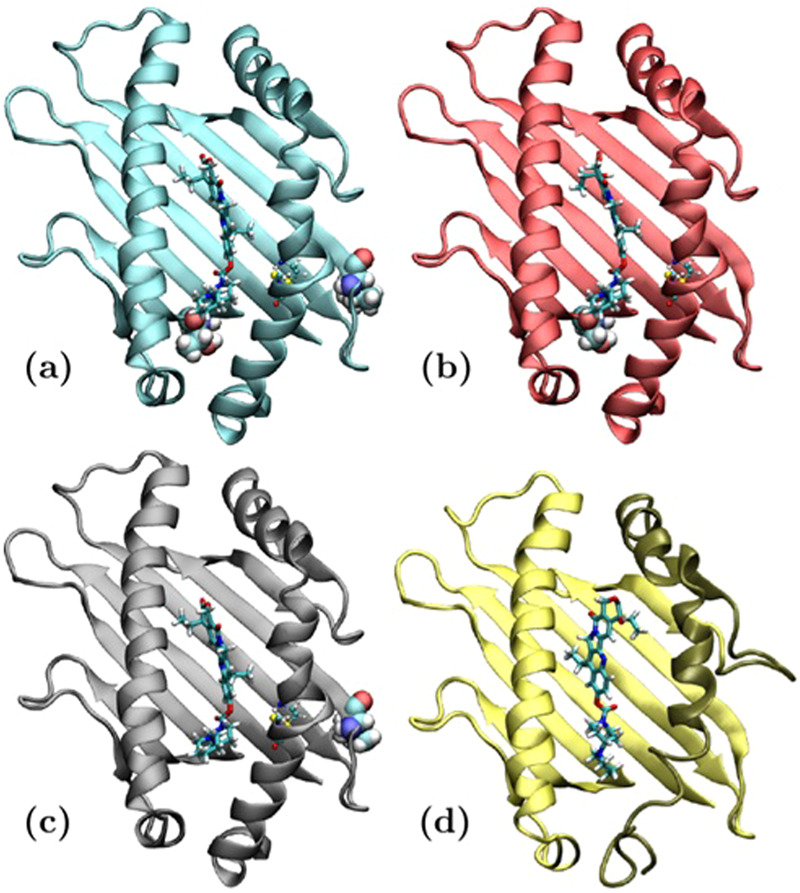
Representative conformation of each HLA-G polymorph and the docked CPT-11 for (**a**) HLA-G*01:01, (**b**) HLA-G*01:03, (**c**) HLA-G*01:04, (**d**) HLA-G*01:05 N. In all cases the S-S bridge forming Cys77 and Cys140 are highlighted. In (**a**–**c**) the mutated amino acids with respect to HLA-G*01:01 (at positions 31 and 110) are represented with their van der Waals spheres, and in (**d**) the substituted 60 aa peptide after Asp 129 is highlighted in a darker colour.

The interaction between HLA-G and CPT-11 was then investigated by computational modelling. For the modelling we considered the α1 and α2 domains of four HLA-G polymorphs: the most common protein HLA-G*01:01 (Fig. 5a), the p.Thr31Ser polymorph HLA-G*01:03 (Fig. 5b), the p.Leu110Ile polymorph HLA-G*01:04 (Fig. 5c), and the mutant HLA-G*01:05 N (Fig. 5d). This latter truncated protein, as a consequence of the changes in the reading frame due to the deletion in codon 130 and the creation of a premature stop codon after 60 residues, does not possess the S-S bridge between Cys77 and Cys140, and the Cys179-Cys235 bridge. The polymorphism in codon 258 p.Thr258Met (defining the HLA-G*01:06) is localized in the α3 domain and was not investigated. The selected polymorphs were modelled by homology modelling.

The irinotecan size and shape are comparable to that of the small peptides presented on the peptide-binding cleft defined by the α1 and α2 domains of MHC class I. Then, the peptide-binding cleft was chosen as putative binding site for the CPT-11. One drug molecule was docked to the cleft of each HLA-G polymorph. The conformation with the lowest score found for CPT-11 in HLA-G*01:01 appears elongated into the pocket (Fig. 5a) similarly to that found for the binding peptides. The molecule is bent towards the α2 domain. The same pose was found in the docking results for polymorphs HLA-G*01:03 (Fig. 5b), and HLA-G*01:04 (Fig. 5c) while it was not found for HLA-G*01:05 N (Fig. 5d). In HLA-G*01:05 N the CPT-11 still sits elongated along the pocket but rotated with respect to the molecular axes to maximise its interaction with the substituted 60 aa peptide after Asp 129. In this case the molecule is bent towards the α1 domain in opposite direction with respect to the other polymorphs. Similarly, we docked CPT-11 metabolites (SN38 and SN38G) to the same binding cleft. Among the generated poses, SN38 does not adopt the same conformation as CPT-11, and it was not found to assume the same pose in G*01:01. In agreement with its reduced size not filling the available cleft space, the shorter SN38 appears most often translated in the cleft with respect to CPT-11 in all polymorphs (Supplementary Fig. 1). The glucuronidation seemed to destabilise SN38G position in the cleft leading to several competing poses: two are the recurring poses one of which with the same conformation and position as that observed for CTP-11 (Supplementary Fig. 2). Overall also the poses observed for SN38 and SN38G appeared little affected by point mutations, and comparable results were found for all polymorphs but G*01:05 N. Thus confirming the different response of the G*01:05 N polymorph also towards CPT-11 metabolites.

**Figure 6:**
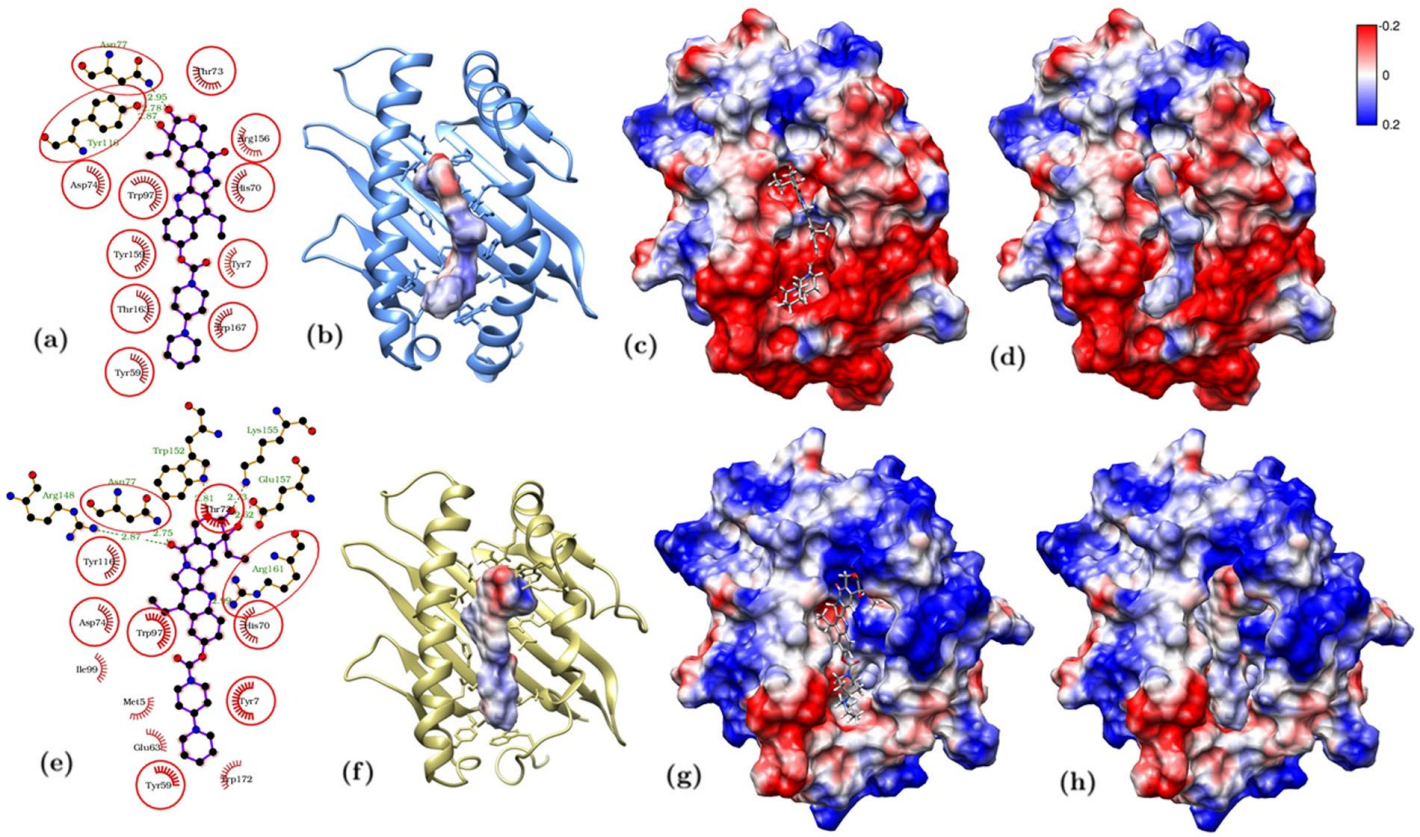
Comparison between representative conformations of CPT-11/HLA-G*01*01 (top panels) and CPT-11/HLA-G*01*5 N (bottom panels): (**a**) schematic diagram of the interaction between CPT-11 and HLA-G*01:01 and surface electrostatic (Coulomb) potential of (**b**) CPT-11, (**c**) HLA-G*01:01, and (**d**) their complementarity; (**e**) schematic diagram of the interaction between CPT-11 and HLA-G*01:05 N and surface electrostatic (Coulomb) potential (kcal·mol^−1^·e^−1^) of (**f**) CPT-11, (**g**) HLA-G*01:05 N, and (**h**) their complementarity. In (**a**,**e**) protein residues interacting with the compounds by Van der Waals interactions are highlighted in red, while hydrogen bonds are indicated by green dotted lines. Interacting residues found also for the other polymorphs are circled.

In the pocket of HLA-G*01:01 (Fig. 6a), as well as in that of HLA-G*01:03 and HLA-G*01:04, CPT-11 interacts with the target protein thanks to 10 hydrophobic interactions and hydrogen bonds with Tyr118 and Asn77 which keep the molecules bound to the pocket. Further, we observe a strong electrostatic contribution. The electrostatic contribution to the binding can be appreciated by calculating the surface electrostatic potential of CPT-11 and HLA-G*01:01 (Fig. 6b,c). Their complementarity (Fig. 6d) confirms the CPT-11 tendency to interact off-target with HLA-G*01:01, HLA-G*01:03, and HLA-G*01:04.

A different, thus related, case is represented by HLA-G*01:05 N (Fig. 6e–h): in this mutant a larger number of interactions keeps CPT-11 bound to the pocket and electrostatic complementarity plays once again an important role. In more details, the hydrophobic interactions increase from 10 to 11 and new hydrogen bonds are formed with α2 Arg148, Arg161, Trp152, Lys155, Glu157, which adds up to the one formed with Asn77 (Fig. 6e) already observed above with the other isoforms (Fig. 6a). The electrostatic landscape is highly affected by the different sequence present in the α2 domain: CPT-11 in fact is observed in a different conformation (Fig. 6f) to match the different landscape of this mutant resenting a positive electrostatic potential along all the substituted chain (Fig. 6g) and partially quenching the negative charge of the protein fragment observed for most common polymorphs (Fig. 6c). Nevertheless CPT-11 in its newly found conformation is capable of high complementarity (Fig. 6g).

**Figure 7:**
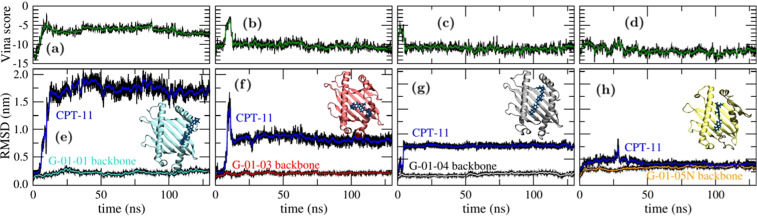
Molecular dynamics simulations. Evolution of Autodock VINA score for the CPT-11 in complex with for (**a**) HLA-G*01:01, (**b**) HLA-G*01:03, (**c**) HLA-G*01:04, (**d**) HLA-G*01:05 N; and root mean squared deviation (RMSD) of CPT-11 and each polymorph backbone in the frame of the respective protein backbone for (**e**) HLA-G*01:01, (**f**) HLA-G*01:03, (**g**) HLA-G*01:04, (**h**) HLA-G*01:05 N. In the insets: end-simulation snapshots.

The presence of a larger binding pattern observed for HLA-G*01:05 N with respect to the most common polymorphs suggests that once the CPT-11 is bound to the protein it could be sequestered and interfere with its activity. To clarify this picture and get some further insight on the effects of mutations on the behaviour of CPT-11 on HLA-G, 130 ns molecular dynamics simulations of each complex in water were run. The complexes binding energy was estimated by scoring the trajectories with the scoring function of Autodock VINA observing relevant interactions, between 5 and 10 kcal/mol, along the whole simulated time for all the polymorphs (Fig. 7a–d). In general CPT-11, while being mobile in the protein binding cleft, does not leave the protein. The effect of point mutations is confirmed to be minor: the molecule is observed to move in the cleft, but never leave it completely (Fig. 7e–g and insets therein). This can be appreciated by following the root mean squared deviation (RMSD) of the CPT-11 atomic positions with respect to the protein backbone. The RMSD is a useful indicator of the average atomic displacement along the simulated time, the lower the RMSD the less mobile are the atoms considered and *viceversa*. The protein backbone RMSD below 0.3 nm observed for most polymorphs indicates that the protein fragments do not undergo dramatic rearrangements even if, as expected, the value reaches 0.5 nm for G*01:05 N suggesting a rearrangement of its frameshifted domain. In this latter case, where CPT-11 is kept into place thanks to several hydrogen bonds which give stability to the complex, only smaller rearrangements are observed in the CPT-11 atom positions with respect to those observed for the most common polymorphs (Fig. 7h).

## Discussion and Conclusions

Current results in the field of immune-oncology underline the relevance of study the immune system actors as a strategy to ameliorate the clinical outcome of patients suffering of a number of cancers. In particular, the recent work of Pagés and colleagues highlighted the importance of using the Immunoscore as new method to stratify patients ensuring a more precise treatment with a better prognostic value in colon cancer^9^. Here we maintain that also the HLA-G expressed by tumour cells could add a new piece to the puzzle involving the mechanism of tumor escape from immunosurveillance and the management of these patients.

The soluble HLA-G expression was here investigated for its possible role as clinical biomarker in mCRC. The inverse relation observed between sHLA-G and CPT-11 AUC levels suggests an irinotecan-based effect on sHLA-G in patient with mCRC. The results found for sHLA-G and number of distant metastastic sites is concordant with the expected increase in the tumor escape phenomenon and to what reported for stage IV mCRC^44^. In patients with CRC the correlation between positive tumor expression of HLA-G and distant metastasis suggested that HLA-G could facilitate tumor immune escape phenomenon, invasiveness, disease progression^10,45,46^. We observed that patients with high sHLA-G levels were associated with diminished irinotecan and then BI levels, with a possible effect on the clinical outcomes of these patients. A correlation between the HLA-G genetic variations and susceptibility and/or clinical outcome of cancers has already been suggested: the expression and predictive relevance of HLA-G expression in 457 patients with primary CRC investigated by immunohistochemical analysis showed that patients with higher levels of HLA-G had a significantly worse survival than those with lower levels, suggesting that a stratification based on the HLA-G expression levels could be independent prognostic factor for patients with CRC^10^. Moreover, in a cohort of 178 Chinese patients with CRC the presence of higher levels of soluble HLA-G in plasma exhibited a significant predictive value of worse survival^47^. High levels of the peripheral sHLA-G are usually associated to a negative prognostic value in CRC. However, the relation of the sHLA-G levels with the prognosis could be considered a controversial issue^44,48,49^. It was raised a critical aspect in the techniques used in CRC for the quantification of HLA-G protein expression that could provide an HLA-G overestimation predominantly in the immunohistochemical studies^50^. Moreover, the HLA-G-targeting antibodies currently available do not allow detecting the expression of all the HLA-G isoforms but only of the most frequent.

Our analysis of the pharmacokinetic parameters suggested a possible mechanism of CPT-11 capture by the HLA-G protein that may alter the interaction of HLA-G with its target receptors, blocking the induction of tolerogenic effects that induces tumor immune escape. This hypothesis was confirmed by testing the association between sHLA-G and CPT-11 by spectrophotometric analysis and molecular modeling. Both confirmed the off-target association between the two compounds. In particular, for the first time, molecular modeling further revealed a different association mechanism for the rarer mutant HLA-G*01:05 N with respect to the most common HLA-G*01:01, HLA-G*01:03, and HLA-G*01:04 HLA-G polymorphs. The presence of HLA-G*01:05 N was associated to the *HLA-G* UTR-2 haplotype related to a lower sHLA-G expression^32^ and it was already demonstrated to correlate with a decreased risk of non-small cell lung cancer in Tunisian population^51^. No specific data for HLA-G*01:05 N in CRC were found in the literature, nevertheless it was demonstrated that *HLA-G* UTR-2 haplotype has a predictive role of neurotoxicity risk in non-metastatic CRC treated with FOLFOX4 (folinic acid/5-fluorouracil/oxaliplatin)^42^. Generally, HLA-G*01:05 N has a low frequency in the world, except for the Middle East populations, in particular North Indian and certain African population as Shona^52^. The evolutive reason of this frequency distribution is not yet known although it has been hypothesized an origin by a founder effect or related to HLA-G*01:05N-allele functions^53^. It has been already reported the HLA-G*01:05 N allele may provide some selective advantage such as avoiding maternal rejection and improving of immune response against some infections in healthy population^53^. Moreover, immune response can also affect cancer development and then the effect of HLA-G*01:05 N allele on cancer is an intriguing issue that needs further investigation. In this HLA-G*01:05 N polymorph we found a larger number of hydrophobic interactions and hydrogen bonds, and also an electrostatic complementarity to keep CPT-11 bound to the pocket. The discovery of this novel HLA-G/CPT-11 interaction suggests two hypothesis of the effect that could be had in patients: (1) it could alter the interaction of HLA-G with its receptors located on the immune system cells, modifying its mechanism of inhibition of immune responses; (2) it could affect the pharmacological effect of the CPT-11, altering its pharmacokinetics. In the first case, this interaction may inhibit the tolerogenic functions of HLA-G against cancer and this could explain good prognostic results found in the literature. In the second hypothesis, we could also have some effects on clinical aspects. A further investigation, also by means of localised mutations, on the interactions between HLA-G variants with CPT-11 metabolites could help to better explain their clinical implication in the treatment of patients with mCRC.

In conclusion, we observed a novel and interesting interaction between irinotecan and HLA-G polymorphs and the more resistant association of irinotecan to HLA-G*01:05 N offer a new opportunity to better evaluate the effect of less common isoforms and maybe to clarify the apparently controversial differences in the effect of HLA-G levels on clinical outcomes reported in several studies conducted in population with different ethnicities and treatments affected by cancer.

The main limit of our study is represented by the small number of patients with mCRC in which the plasmatic concentration of sHLA-G was retrospectively tested. Considering that the inverse correlation between the PK parameters and sHLA-G levels was observed in a small subgroup of patients, our results should be viewed as hypothesis generating to be validated in a further work though a correlation analysis with clinical data such as response to treatment and survival, with a proper sample size of patients affected by mCRC and homogeneously treated with irinotecan-based regimen. However, the preliminary data concerning the off-target CPT-11/HLA-G association sustain this finding. Indeed, one important strength of this study, is that, to our knowledge, this is the first exploratory investigation about the unspecific interaction between HLA-G and a chemotherapeutic drug currently used to treat patients with different advanced malignancies, including mCRC.

## Methods

### Patients clinical data and treatment

Clinical data, genomic DNA from blood samples and plasma samples were obtained from patients diagnosed with mCRC prospectively collected by Toffoli and colleagues for two previous published studies^43,54^. Inclusion criteria for our retrospective study was defined by plasma sample availability: 25 samples derived from the first study^43^ and 15 from the genotype-driven phase I study published in 2010^54^. All the plasma samples analyzed in this study were collected prior to FOLFIRI administration. About the second study^54^, patients were selected for UGT1A1*1/*1 and UGT1A1*1/*28 genotypes and treated with FOLFIRI regiment: irinotecan was administered at doses higher than the standard 180 mg/m2 starting from 215 mg/m 2 with a 20% increase every-2-weeks dose until a dose of 370 mg/m2 for UGT1A1*1/*28 patients and of 420 mg/m2 for UGT1A1*1/*1 patients, with unchanged dose of infusional FU/LV. FOLFIRI was discontinued because of disease progression, intolerable side effects, patient refusal, or physician assessment. According to the eligible criteria of the study^54^, patients who carried the UGT1A1*28/*28 were excluded. The institutional review board of each participating institution and the Ethics Committee of the Centro di Riferimento Oncologico (CRO) of Aviano had approved the study protocol (CRO-26-2002 and CRO-34-2005), and all patients signed a written informed consent for a genetic analysis before entering the study^43^. All the analyses were performed in accordance with the Declaration of Helsinki principles.

### Assay for plasma sHLA-G

We analyzed the 40 available EDTA plasma samples using the sHLA-G enzymed-linked immunosorbent assay (ELISA) kit (BioVendor–Laboratorní medicína a.s., Brno, Czech Republic), a sandwich enzyme immunoassay for the quantitative measurement of sHLA-G, according to the manufacturer’s instructions. This ELISA assay detects both membrane shedded HLA-G1 and soluble HLA-G5. The absorbance was measured by Infinite F200 PRO (TECAN, Männedorf, Switzerland) at 450 nm with the reference wavelength set to 630. All samples were assayed in duplicate and the final sHLA-G concentrations were determined from a five-point standard curve using dilutions of calibrator (7.81, 15.63, 31.25, 62.5, and 125 U/ml) purchased by the kit as standard reagent. Results were expressed as Units/milliliter (U/mL).

### Statistical analysis

The plasma levels of sHLA-G were evaluated in relation to clinical variables, patient’s genotype and pharmacokinetic (PK) parameters: comparison between two groups was performed using the non-parametric Mann-Whitney test and between three or more groups with the Krustal-Wallis test. Analyses were done with GraphPad Prism 3.02 (GraphPad Software). All the statistical analyses were two-tailed and a *p* < 0.05 was considered statistically significant.

### Chemical and reagents

CPT-11 hydrocloride (Sigma-Aldrich, Saint Louis, MO, USA), with a molecular weight of 677.18 g/mol, was dissolved in dimethylsulfoxide (DMSO) solution at a stock concentration of 1.6 mM and then diluited with milliQ water to the different work concentrations used in the experiment. CPT-11 hydrocloride were kept at 4 °C and fresh solution were prepared every 3–6 hours to consider the hydrolysis of the lactone. The stock solution was prepared at a concentration of 1 mg/ml (corresponding to 15 μM) in milliQ water. Recombinant glycosylated HLA-G protein expressed in mammalian cells with N-terminal with a 6xHis-tagged (MyBioSource, San Diego, CA, USA) was lyophilized from a 0.2 μm filtered 10 mM Tris-HCl, 1 mM EDTA, pH 8.0. The HLA-G was reconstituted in 100 μl of deionized sterile water (milliQ) to a final concentration of 1 mg/ml, corresponding to a concentration of 25 μM, according to the datasheet instructions. After reconstitution, for short (long) term storage all proteins were kept at 4 °C (-20 °C).

### UV-Vis absorption

UV-Vis absorption spectra were recorded over a wavelength range of 200–800 nm. Absorption spectra of blank, HLA-G and CPT-11 were recorded on the Cary Eclipse UV-Vis Spectrophotometer (Agilent Technologies, Santa Clara, CA, USA) and were the mean of 4 spectra acquisitions. The final concentrations analyzed were 0.01, 0.05 and 0.1 μM for HLA-G, and 0.01, 0.05, 0.1, 1, 5, 10, 25, 50 μM for CPT-11. Quartz cuvettes with a pathlength of 10 mm and a volume of 700 μl (Hellma-Analytics, Müllheim, Germany) were used. We calculated the molar concentration though the Beer-Lambert law^55^ using the theoretical extinction coefficient obtained from the primary sequence of the proteins calculated with the ExPASy ProtParam tool (http://www.expasy.org/tools/pi_tool.html).

### Fluorescence

Fluorescence emission spectra of blank, HLA-G and CPT-11 were measured using the Cary Eclipse Fluorescence Spectrophotometer (Agilent Technologies, Santa Clara, CA, USA). The concentrations analyzed were 0.01, 0.05 and 0.1 μM for HLA-G, and 1, 5, 10, 15, 20 μM for CPT-11. Emission spectrum of the molecules was recorded from 280 to 550 nm and from 365 to 550 nm at an excitation wavelength of 275/280 and 360 nm, respectively. The emission and excitation slits widths were set at 2.5 and 5 nm with a photomultiplier tube (PMT) detector voltage of 800 and 500 Volts, respectively. Fluorescence quartz cuvettes with a pathlength of 3 mm × 3 mm and a volume of 45 μl (Hellma-Analytics, Müllheim, Germany) were used. Using the same instrument setting, we performed a titration assay measuring the fluorescence emission spectra of HLA-G and CPT-11, adding to a fix concentration (0.1 μM) of HLA-G a certain amount of CPT-11 each time, changing concentrations of CPT-11 in solution to 1, 5, 10, 15, 20 μM during the interaction. Data were fitted using Microsoft Excel Solver together with SolverAID, the latter part of R. de Levie MacroBundle (v.12 May 2012).

### Homology modelling

We chose HLA-G sequences corresponding to the α1 and α2 domains (aa 2–182) of HLA-G*01:01, HLA-G*01:03, HLA-G*01:04, HLA-G*01:05 N coding alleles from http://hla.alleles.org/data/hla-g.html (NCBI Reference Sequence: NP 002118.1 or HLA00939 G*01:01:01:01 in ftp://ftp.ebi.ac.uk/pub/databases/ipd/imgt/hla/fasta/G_prot.fasta). All modelled sequences started with SHSMRYFSAAV (thus discarding the first 26 residues with respect to the sequences linked above), and the first amino acid of the chain was labelled as Ser2. Chain A of structure 1YDP^56^ was used as a template for all the mutants. The most common sequence G*01:01 (corresponding to the experimental sequence) presents three mutations with respect to the 1YDP: S42C, I110L, R115Q. All the sequences but one were reconstructed by alignment and fitting with DeepView - Swiss-PdbViewer 4.1 and G*01:05 N was built with Swiss Model. All the models were minimized by first minimizing the protein side chains alone then whole protein with the AMBER99SB force field as implemented in GROMACS package v. 2016.1.

### Molecule preparation and docking

The irinotecan initial conformation was protonated, minimized with AM1 method as implemented in MOPAC 2016. The molecule was docked into each protein representative conformation prepared as described above. The system was prepared with AutoDock tools, and docked with AutoDock. We used Lamarckian Genetic Algorithm with docking box (26.250 × 26.250 × 41.25)Å centred on the alpha carbon of Tyr7 with 0.375 Å grid spacing. The docking was performed with 1000 runs and 2,500,000 maximum numbers of evaluations and standard parameters. End-conformations were clustered and the representative conformation of each cluster was chosen. Comparison between 1YDP crystal structure and its redocked ligand had RMSD 0.9 nm, thus validating the method. End-conformations of HLA-G*01:03, HLA-G*01:04, HLA-G*01:05 N were further visually inspected for the occurrence of the first ranked irinotecan pose found for HLA-G*01:01. 2D ligand-protein interaction diagrams were generated with LigPlot+. Representative conformations were thus minimised by first minimizing the protein side chains alone, then whole protein and finally the whole system by constraining selected portions of the system as reported below. Surface electrostatic potentials in the form of Coulomb potentials with distance dependent dielectric constant ε = 80.0 (water), at 1.4 Å from the surface, and 298 K as implemented in UCSF Chimera.

### Molecular dynamics simulations

Each selected HLA-G/irinotecan complex was minimised by constraining selected portions of the system and progressively releasing them. The complex was then hydrated in a cubic box with a tip3p water layer of 0.7 nm and the whole system was minimised again. AMBER99SB force field was used with ligand topologies built with Antechamber. Antechamber parameters were validated by comparing the molecular coordinates after minimisation with respect to the semiempirical QM calculation (RMSD = 0.013 nm). NVT and NPT equilibrations were performed for 100 ps, followed by 130 ns NPT production run at 300 K with 2 fs iteration time step, Verlet integrator, LINC constraint, and periodic boundary conditions. All the simulations and their analysis were run as implemented in the GROMACS package v. 2016.1. Simulations were run on Marconi (CINECA, Italy).

## Supplementary information

Supplementary information.
