# Supplementary material for: Soluble HLA-G expression levels and HLA-G/irinotecan association in metastatic colorectal cancer treated with irinotecan-based strategy
Source: Sci Rep. 2020 May 29;10:8773. doi: 10.1038/s41598-020-65424-z (PMC7260212; doi:10.1038/s41598-020-65424-z)
Supplement: Supplementary file 1 — Supplementary information. [file 41598_2020_65424_MOESM1_ESM.pdf]

Supplementary Information

Soluble HLA-G expression levels and HLA-G/irinotecan association in metastatic colorectal cancer treated with irinotecan-based strategy

Lucia Scarabel[1], Marica Garziera[1], Sara Fortuna[2,*], Fioretta Asaro[2], Giuseppe Toffoli[1], Silvano Geremia[2]

[1]Experimental and Clinical Pharmacology Unit, Centro di Riferimento Oncologico (CRO), IRCCS, 33081 Aviano, Italy

[2]Department of Chemical and Pharmaceutical Sciences, University of Trieste, Via L. Giorgieri 1, 34127 Trieste, Italy

*Correspondence: e-mail: s.fortuna@units.it; web: www.sarafortuna.eu; Tel: +39-040-5583671/3922

Fitting of HLA-G and Irinotecan titration assay data

The addition of CPT-11 to a solution of HLA-G is ruled by the equilibrium:

(1) $$\text{HLA-G} + \text{CPT-11} \rightleftarrows \text{HLA-G:(CPT-11)}$$

with association constant K_a

(2) $$K_a=[\text{HLA-G:(CPT-11)}]/([\text{HLA-G}][\text{CPT-11}])$$

The addition of CPT-11 to a solution of HLA-G induces a quenching of the protein fluorescence and a significant increase of the fluorescence signal of CPT-11. The fluorescence emission F at 430 nm was considered as sum of the fluorescence of bound and free CPT-11. The fluorescence of each species can be safely considered linearly dependent on concentration at high dilution of the reagents:

(3) $$F= F^{o}_{f} [\text{CPT-11}] + F^{o}_{b}[\text{HLA-G:(CPT-11)}]$$

where F^{o}_{f} is the emission intensity of free CPT-11 measured experimentally (17 a.u./μM) and F^{o}_{b} is that of CPT-11 bound to HLA-G which is a fitting parameter along with the Ka.

The fitting parameters were obtained from fluorescence emissions at 430 nm (mean of 4 scans) of HLA-G 1 μM with the addition of CPT-11 at 0.1, 0.2, 0.4, 0.8, 1, 2, 4, 8 μM. The non-linear fitting with EXCELL solver and solverAID macros led to F^{o}_{b} = (800±60) a.u./μM and K_a=(0.54±0.17)μM with adjusted coefficient of determination $\bar{R}^2$=0.986.

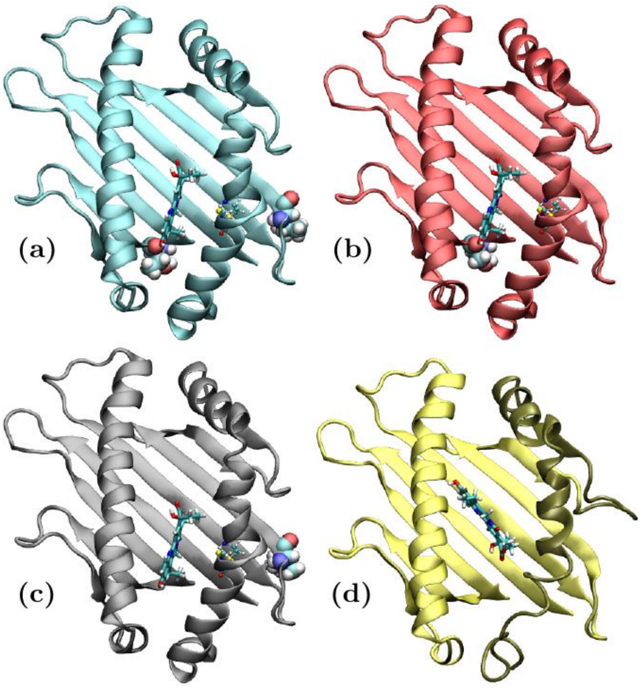

Supplementary Figure 1. Representative conformation of each HLA-G polymorph and the docked SN38 for (a) HLA-G*01:01, (b) HLA-G*01:03, (c) HLA-G*01:04, (d) HLA-G*01:05N. In all cases the S-S bridge forming Cys77 and Cys140 are highlighted. In (a-c) the mutated amino acids with respect to HLA-G*01:01 (at positions 31 and 110) are represented with their van der Waals spheres, and in (d) the substituted 60 aa peptide after Asp 129 is highlighted in a darker color.

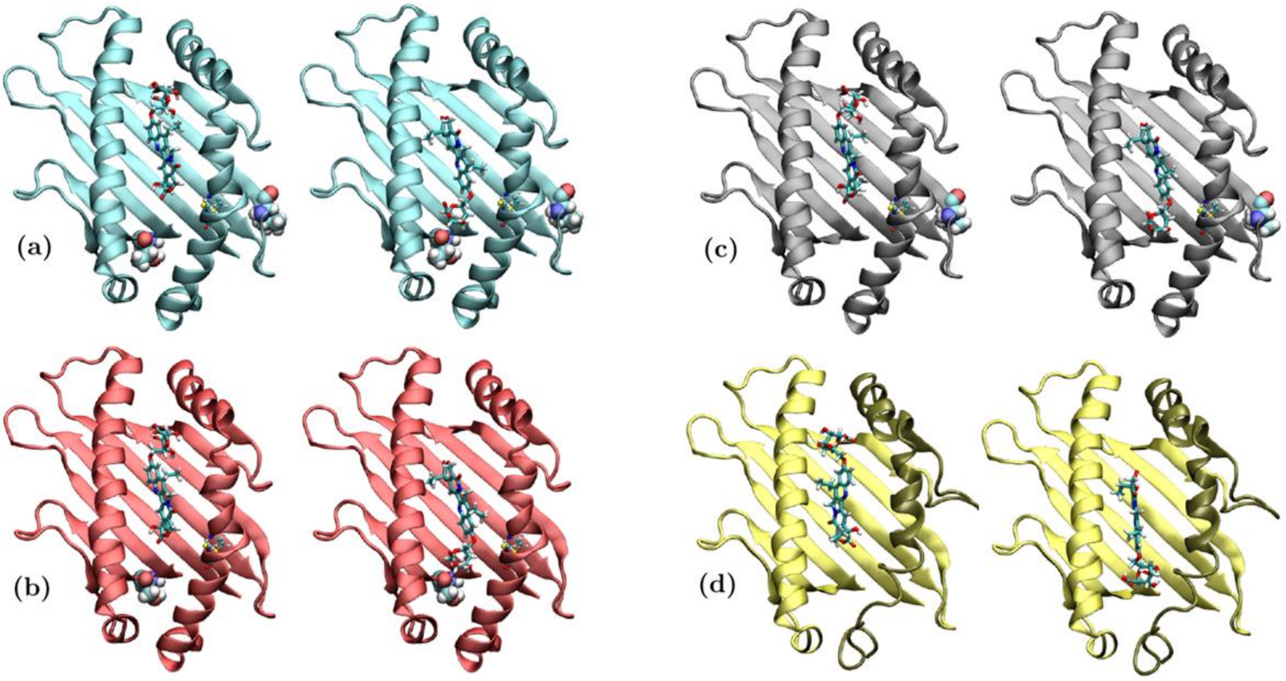

Supplementary Figure 2. Representative conformation of each HLA-G polymorph and the docked SN38G for (a) HLA-G*01:01, (b) HLA-G*01:03, (c) HLA-G*01:04, (d) HLA-G*01:05N. In all cases the S-S bridge forming Cys77 and Cys140 are highlighted. In (a-c) the mutated amino acids with respect to HLA-G*01:01 (at positions 31 and 110) are represented with their van der Waals spheres, and in (d) the substituted 60 aa peptide after Asp 129 is highlighted in a darker color.
